# Molecularly imprinted nanoparticles for pathogen visualisation†

**DOI:** 10.1039/d2na00913g

**Published:** 2023-03-17

**Authors:** Jaroslava Bezdekova, Francesco Canfarotta, Fabiana Grillo, Hasan Yesilkaya, Marketa Vaculovicova, Sergey Piletsky

**Affiliations:** a Mendel University in Brno Zemedelska 1 Brno 613 00 Czech Republic; b MIP Discovery, The Exchange, Colworth Science Park Sharnbrook MK44 1LQ UK; c University of Leicester University Rd Leicester LE1 7RH UK fg90@leicester.ac.uk

## Abstract

Saccharides displayed on the cell surface of pathogens play critical roles in many activities such as adhesion, recognition and pathogenesis, as well as in prokaryotic development. In this work, we report the synthesis of molecularly imprinted nanoparticles (nanoMIPs) against pathogen surface monosaccharides using an innovative solid-phase approach. These nanoMIPs can serve as robust and selective artificial lectins specific to one particular monosaccharide. The evaluation of their binding capabilities has been implemented against bacterial cells (*E. coli* and *S. pneumoniae*) as model pathogens. The nanoMIPs were produced against two different monosaccharides: mannose (Man), which is present mainly on the surface of Gram-negative bacteria, and *N*-acetylglucosamine (GlcNAc) exposed on the surface of the majority of bacteria. Herein, we assessed the potential use of nanoMIPs for pathogen cell imaging and detection *via* flow cytometry and confocal microscopy.

## Abstract 2

Fluorescent image (left) and graphical representation (right) of saccharides displayed on the cell surface bound by fluorescent nanoMIPs.

## Graphical abstract

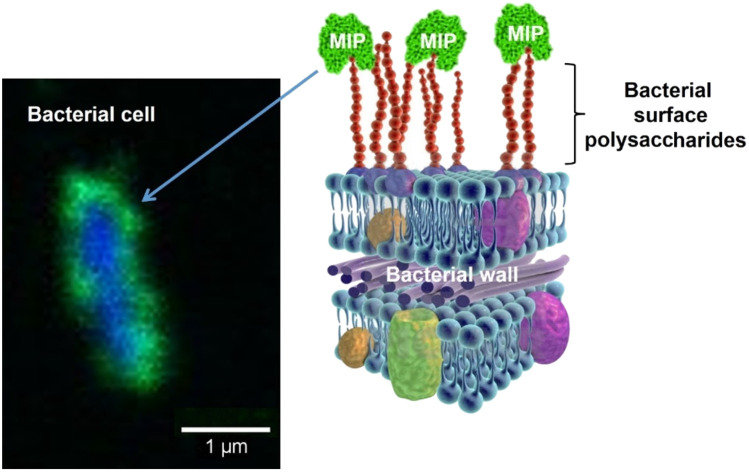

## Introduction

It is well-known that the surface of pathogens (*e.g.* bacteria, viruses, fungi, *etc.*) is rich in glycol-conjugates such as capsules, lipopolysaccharides and peptidoglycans. Knowledge of the surface carbohydrates and their variations is desirable in understanding many biological processes such as cellular recognition, adhesion and the immune response function at the molecular level.^1,2^ A further understanding of the surface glycan's composition in pathogens can provide the basis for the design of saccharide-based vaccines, diagnostic agents or immune stimulators.^3^ Despite it being known that pathogenic glycosylation influences a range of vital processes, their role has not been explored in-depth so far.

Lectins are proteins obtained from animals or plants that recognise and bind carbohydrates. Due to this feature, lectins are currently considered the gold standard for the verification of the presence of surface monosaccharides in many diagnostic applications.^4–6^ However, lectins present some disadvantages, such as a relatively low affinity. Furthermore, they are generally not selective for one specific monosaccharide.^7^

In the present proof-of-concept work, we report the development of fluorescent polymeric binders – molecularly imprinted nanoparticles (nanoMIPs) – prepared by solid-phase imprinting as artificial lectins against Man and GlcNAc, for the detection/visualization of pathogens (specifically for *E. coli* and *S. pneumoniae*). NanoMIPs are synthetic binders produced *via* a self-assembly process, which involves a template molecule (*i.e.* the molecule to be detected) and monomers with specific functional groups capable of interacting with said template. Following polymerisation and removal of the template, binding sites specific for the imprinted molecule are created within the polymer matrix, which will then retain a “molecular memory”, allowing the polymer to specifically rebind the molecule imprinted in the first place. NanoMIPs exhibit binding affinity and selectivity in line with their natural counterparts, such as antibodies or lectins. Some of the great advantages of these synthetic materials in comparison to their natural counterparts are their robustness,^8,9^ fast turnaround times (a typical nanoMIP can be produced and characterised within 2–4 weeks), complete control over the surface chemistry for attachment on surfaces, the possibility of simple fluorescent labelling during the synthesis process without loss of recognition abilities, and the possibility to imprint virtually any compound. Furthermore, the development of such artificial binders does not involve the use of animals.^10^ The artificial lectins herein presented can be used to provide information on the pathogen cell surface and potentially lay the foundation for the development of diagnostics assays specific for bacteria.

To date, several nanoMIPs imprinted for the whole bacterial cell or one specific bacterial cell protein/peptide have been produced (Tables S1 and S2†). However, imprinting of large structures (such as cells) may cause difficulties in their removal from the polymerised system. In order to overcome this, Vaculovicova *et al.* prepared magnetic particles with molecularly imprinted polymer (MIP) coating selective for the whole cells of *S. aureus*. In the bacterial elution step, removal of bacterial cells was accomplished by magnetic separation; however, some cells were found to be buried in the polymeric layer and this resulted in a reduction of the overall binding capacity of the MIPs.^11^ Moreover, the relatively large binding sites created by imprinting the whole cells may lead to a reduction of the selectivity profile, since other bacteria can have similar surface structures to those of the imprinted cells. In another example, Shen *et al.* synthesized MIP particles capable of rebinding the whole cell of *E. coli*, however, the selectivity data indicated a relatively high degree of cross-reactivity with other bacteria such as *Lactobacillus* sp.^12^ The same issue was solved in a study published by Yilmaz *et al.* where MIP layer selective for *E. coli* has cross-reactivity against *Bacillus* sp.^13^

These cross-reactivity issues can potentially be solved by imprinting specific bacterial proteins or the application of the epitope imprinting approach, which is based on imprinting a small fragment of the target protein. Such an improvement in selectivity is linked to the low probability of finding small protein fragments on other bacteria. Therefore, it is clear how paramount it is to appropriately choose the epitope/protein to be imprinted. Wu *et al.* created MIP against *S. aureus* protein A (adsorbing capacity – 10^2^ to 10^3^ CFU g^−1^). In this case, the recognition ability against various competitive bacteria was improved (adsorbing capacity on *E. coli* and *S. thermophilus* are under 10 CFU g^−1^).^14^ An example of such enhancement in selectivity given by the epitope approach is the work of Singh *et al.*, who developed nanoMIPs for an epitope of *Mycobacterium leprae*. These nanoMIPs specifically recognised the imprinted epitope among various other epitopes and proteins with high selectivity.^15^ However, it is worth noting that cross-reactivity between different bacteria was not investigated.

In 2015, monosaccharides were used for the first time as targets for cell and tissue imaging.^16^ Since then, several other works focused on this topic have been published (Table S3†). In one such example, Shinde *et al.* employed sialic acid moieties as templates to produce nanoMIPs; these demonstrated to enable selective staining of different cell lines, proportionally to the sialic acid expression levels.^17^ In a similar attempt, Demir *et al.* developed fluorescent nanoMIPs specific for glucuronic acid, which demonstrated their potential for biotargeting and bioimaging of hyaluronan on fixated human cervical cancer cells.^18^ However, the approach presented in this work – based on the detection of surface sugars by nanoMIPs in bacteria – is completely novel. Natural mannose-binding lectin (MBL) are produced by the innate immune system in order to recognise repeating sugar arrays of microbial surfaces, in order to fight pathogen infections. These calcium-dependent lectins typically coordinate with specific monosaccharides such as *N*-acetyl-d-glucosamine (GlcNAc), mannose (Man), *N*-acetyl-mannosamine and fucose.^4^ Some bacteria, mostly Gram-negative, and other microorganisms, like fungi and yeasts, possess mannose-rich glycans that function as pathogen-associated molecular patterns, which are recognised by the immune system.^19^ GlcNAc is the main component of peptidoglycan of which bacterial cell walls of both Gram-positive and Gram-negative bacteria are composed. A GlcNAc polymer is also present on the cell surface and has been found to be important for biofilm formation in a wide range of bacteria, however, the amount of its surface expression may vary from bacterium to bacterium.^20^ In this work, we describe the use of a solid-phase based approach to produce selective artificial lectins (*i.e.* nanoMIPs) against mannose and GlcNAc, and their evaluation as potential tools to identify and distinguish different classes of bacteria (or more generically microorganisms) based on the presence or absence of the chosen bacterial surface saccharides.

## Results and discussion

**Fig. 1:**
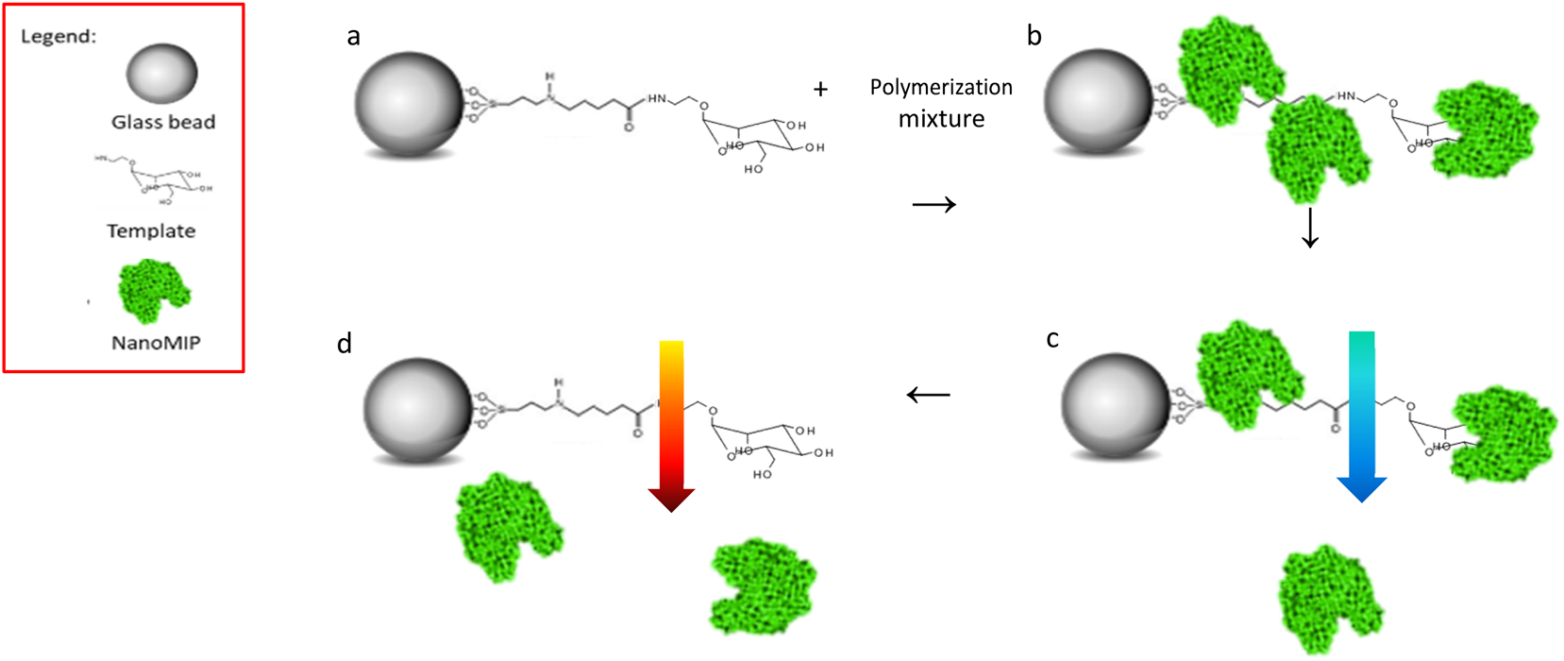
Scheme of solid phase imprinting of monosaccharide nanoMIPs, (a) glass beads with Man immobilised mixed to polymerisation mixture; (b) UV polymerisation, nanoMIPs generation; (c) low temperature washes, removal of low affinity nanoMIPs; (d) high temperature elution, high affinity nanoMIPs' collection.

NanoMIPs were prepared by using an innovative solid-phase approach (Fig. 1), which is based on the covalent immobilization of the template (Man or GlcNAc) onto the surface of a solid carrier (in this case, glass beads). The glass beads are then incubated with a mixture of monomers. Subsequently, a UV-initiated polymerization is carried out. The solid phase is then used as an affinity separation column, such that unreacted monomers and low affinity nanoMIPs are first eluted at room temperature – whilst the high affinity nanoMIPs still remain on the beads. Then, the temperature of the solvent is raised and high affinity nanoMIPs are selectively harvested.^21,22^

The production of nanoMIPs in organic solvents (as presented here) is advantageous for imprinting of small molecules (MW < 500 Da), because it avoids the disruptive effects of water (owing to its hydrogen bonding capacity), of particular importance when imprinting molecules that possess a limited number of interaction points with the monomer.^23^

Control nanoMIPs were prepared using the same process and monomer composition, only using an unrelated target as template for imprinting.

### NanoMIP size and affinity

The size of both nanoMIPs was determined by dynamic light scattering (DLS) using a Zetasizer Nano from Malvern Instruments Ltd (Malvern, UK). An average hydrodynamic size of 55.56 ± 6.13 nm for Man nanoMIPs and 111 ± 7.46 nm for GlcNAc nanoMIPs (both in water) was calculated (DLS graph in Fig. S1†). The homogeneity of the prepared nanoMIPs is demonstrated by the relatively low polydispersity (PdI 0.15 – Man-nanoMIP, PdI 0.21 – GlcNAc-nanoMIP). Scanning electron microscopy (MIRA3 LMU, Tescan, Brno, CZ) was employed to determine shape and size of the prepared nanoMIPs (ESI, S4A and S4†). The affinity of the produced nanoMIPs was verified by surface plasmon resonance (SPR). In particular, an immobilisable version of the target (2-aminoethyl-alfa-mannopyranoside) was used to modify the surface of the SPR gold chip. The target was immobilised *via* EDC/NHS chemistry, whilst the other three channels were used as controls. Then, nanoMIPs were injected in all four channels in PBS. Software analysis revealed a *K*_D_ of 1.6 nM for the nanoMIPs imprinted for mannose (Man-nanoMIPs). A similar approach was used to assess the affinity of GlcNAc nanoMIPs: the target molecule was immobilised on the surface of chips bearing primary amines *via N*,*N*′-disuccinimidyl carbonate chemistry. GlcNAc nanoMIPs were then injected in PBS and software analysis revealed a *K*_D_ of 8.5 nM.

### Interaction between bacteria and nanoMIPs

*E. coli* and *S. pneumoniae* were chosen as model systems representative of Gram-negative and Gram-positive bacteria, respectively. In order to assess the binding performance of the developed nanoMIPs, the aforementioned two bacteria were incubated with nanoMIPs which were used as synthetic lectins for the determination of Man and GlcNAc.

GlcNAc is a part of the peptidoglycan which is a key component of bacterial cell wall which is found on the surface of each bacterium.^20^ This monosaccharide is often present in its polymeric form on the cell surface and has been found to be of significant importance for biofilm formation in a wide range of bacteria including *E. coli* and *S. pneumoniae*.^24,25^ Therefore, it was expected that GlcNAc selective nanoMIPs would interact with both bacteria.

On the other hand, Man is a sugar that occurs mainly on Gram-negative bacteria, as confirmed by Tichaczek-Goska in her work where Man is in its polymerised form, mannan, in various Gram-negative bacterial species including *E. coli*.^26^ Zamze and her team investigated bacterial capsular polysaccharides of different bacteria including *S. pneumoniae* finding many amino-sugars such as *N*-acetyl-d-mannuronic acid d-Gal, d-Glc, and l-Rha present on the surface, whereas d-Man is not found.^27^ Therefore, in this case, it was expected that Man selective nanoMIP will interact only with *E. coli*.

**Fig. 2:**
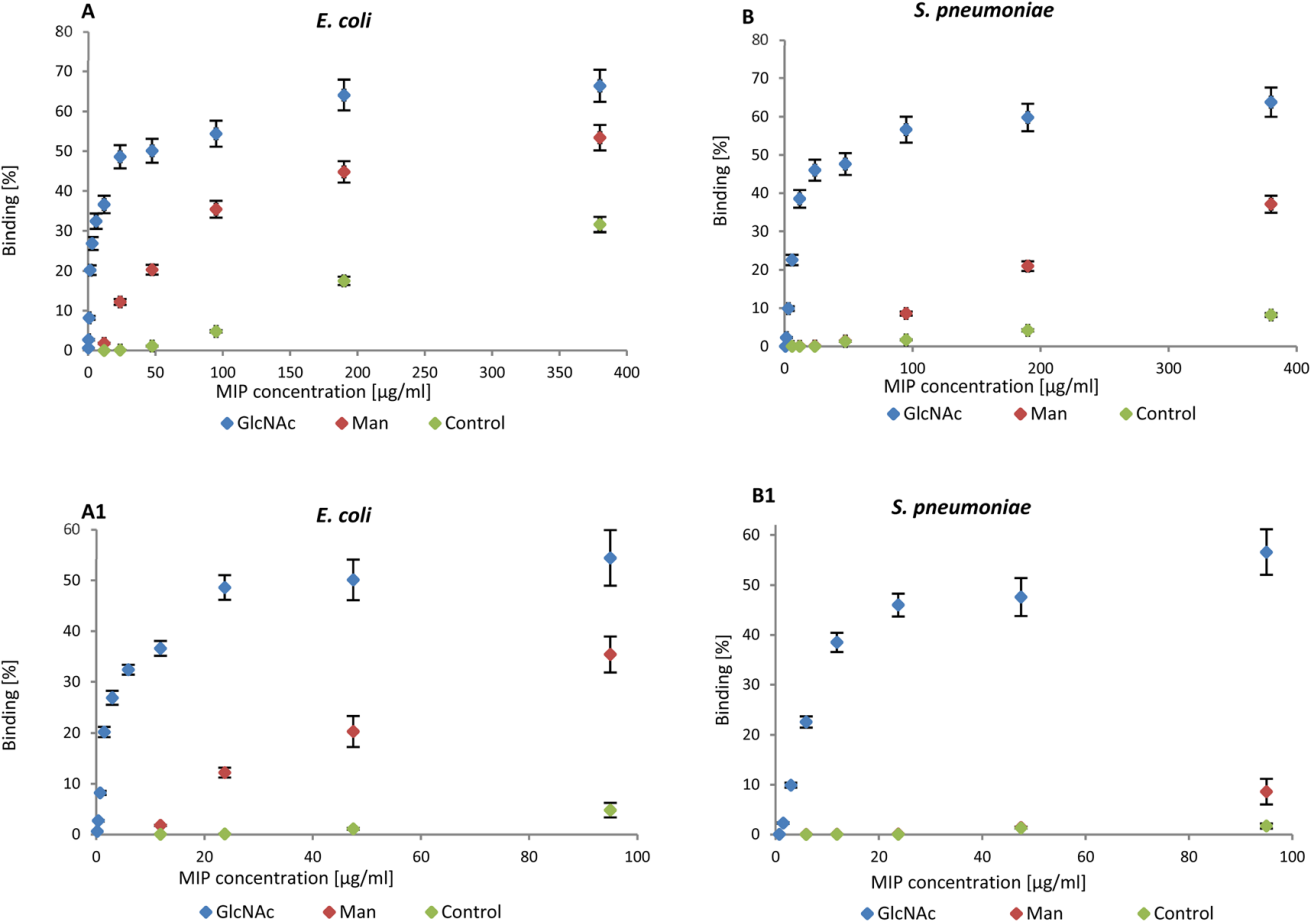
(A) Binding of nanoMIPs to Gram-negative and (B) positive bacteria in the range 0–380 μg mL^−1^ assessed by flow cytometry. A1 and B1 are related to the range 0–95 μg mL^−1^.

NanoMIPs binding to bacteria were observed by flow cytometry, confirming the data in literature. The graphs reported below summarise the data collected by flow cytometry (Fig. S2 and S3†). NanoMIPs imprinted for GlcNAc (blue) were found to bind both *E. coli* (Gram-negative) and D39_*S. pneumoniae* (Gram-positive) as shown in Fig. 2A and B.

Man-nanoMIPs (red) specifically recognise *E. coli* (Fig. 2A) and the binding to *S. pneumoniae* is significantly lower (Fig. 2B). The binding percentage (calculated as nanoMIPs binding the total amount of bacteria in percentage) between GlcNAc-nanoMIPs and *S. pneumoniae* ranges from 0–57% binding at nanoMIPs concentrations between 0.7–95 μg mL^−1^ (Fig. 2B1) appearing to reach a plateau at 64% binding with the highest concentration of nanoMIPs tested (380 μg mL^−1^). Similar observations can be made for the binding between GlcNAc-nanoMIPs and *E. coli* (Fig. 2A).

At low concentrations of Man-nanoMIPs (6–95 μg mL^−1^) (Fig. 2B1) no binding is observed between Man-nanoMIPs and *S. pneumoniae* (no Man on the surface), whereas in the same range of concentrations of Man-nanoMIPs, the binding to *E. coli* is consistently increasing (0–35%) (Fig. 2A1). When the concentration of Man-nanoMIPs is relatively high (95–380 μg mL^−1^) some degree of non-specific binding (NSB) to *S. pneumoniae* is observed (maximum value of 37%). NanoMIPs imprinted for a small unrelated compound are used as control and some low NSB is detected with respectively 8 and 31% binding for *S. pneumoniae* and *E. coli* when the concentration of nanoMIPs is relatively high (380 μg mL^−1^).

Even though at high concentrations of nanoMIPs some NSB is observable, it is, however, negligible when compared to the specific interaction observed between Glc-NAc and the corresponding nanoMIPs. The binding interaction to both *E. coli* and *S. pneumoniae* is around 50% even when the concentration of nanoMIPs is 48 μg mL^−1^ compared to the control nanoMIP respectively (1.1% and 1.3%) to *E. coli* and *S. pneumoniae*.

### Binding analysis *via* surface plasmon resonance (SPR)

**Fig. 3:**
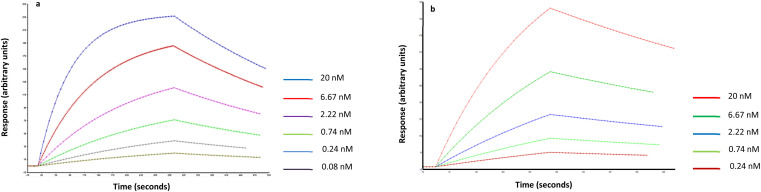
(a) Fitted data related to Man-nanoMIPs and (b) GlcNAc nanoMIPs in PBS (1 : 1 Langmuir binding model with baseline drifting).

The affinity and selectivity of the produced Man-nanoMIPs was verified by surface plasmon resonance (SPR). An immobilisable version of the target (2-aminoethyl-alfa-mannopyranoside) was used to modify the surface of the SPR gold chip. The target was immobilised *via* EDC/NHS chemistry, whilst the other three channels were used as controls. Then, nanoMIPs were injected in all four channels in PBS. Software analysis revealed a *K*_D_ of 1.6 nM for the Man-nanoMIPs. A similar approach was used to assess the affinity of GlcNAc-nanoMIPs: the target molecule was immobilised on the surface of chips bearing primary amines *via N*,*N*-disuccinimidyl carbonate chemistry. GlcNAc-nanoMIPs were then injected in PBS and software analysis revealed a *K*_D_ of 8.5 nM (Fig. 3A and B).

### Interaction between lectins and nanoMIPs

Lectins are proteins that are instrumental in innate immunity as opsonins. As mentioned above, mannose-binding lectins (MBL) are naturally present in the human body and are able to specifically recognise carbohydrates. MBLs bind to a range of sugars including GlcNAc and Man.^4^ In this work concanavalin A (ConA) – which has affinity for terminal α-d-mannosyl and terminal GlcNAc – was chosen as a control to confirm the presence of carbohydrates in bacteria.^28^

**Fig. 4:**
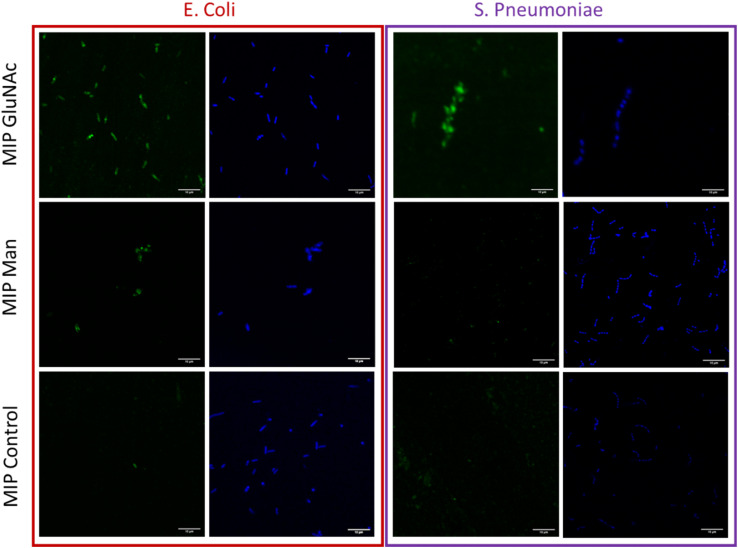
Fluorescence confocal microscopy images of immobilized bacteria (*E. coli* and *S. pneumoniae*) stained with 4 μM DAPI – blue colour. Bacteria treated with nanoMIPs selective for different monosaccharides (Man, GlcNAc) and with control nanoMIPs doped with fluorescent monomer – green colour, scale bar (10 μm) is uniform for all images.

In order to aid in its visualisation, ConA was labelled with a fluorescent dye (Alexa 647) and binding between bacteria and ConA was assessed by using flow cytometry. From measured data (Fig. S5†) it was observed that the amount of bound ConA was higher in the case of *E. coli*. These data confirm that *E. coli* bears higher amount of Man molecule on their surface compared with *S. pneumoniae* (Fig. 4).

### The visualization of binding between nanoMIPs and bacteria

Flow cytometry analysis was used to assess and quantify the binding performance of Man and GlcNAc-nanoMIPs to bacterial surface monosaccharides. To further qualitatively assess the binding of fluorescent Man/GlcNAc-nanoMIP, confocal microscopy was performed. The bacterial nucleoid was stained with DAPI (blue), whilst fluorescent labelled nanoMIPs showed green emission. It was observed that GlcNAc-nanoMIPs bind both *E. coli* and *S. pneumoniae*. Whereas Man-nanoMIPs interacted with *E. coli*, but not with *S. pneumoniae*, which is in agreement with the results obtained by flow cytometry and also with literature.^27^ NanoMIPs imprinted for a small unrelated compound served as control and interacted neither with *E. coli* nor *S. pneumoniae*.

## Materials and methods

Sodium hydroxide (NaOH), methacrylic acid (MAA), ethylene glycol dimethacrylate (EGDMA), trimethylolpropane trimethacrylate (TRIM), pentaerythritol tetrakis(3-mercaptopropionate) (PETMP), *N*,*N*-diethyldithiocarbamic acid benzyl ester (INIFERTER), *N*-(3-aminopropyl) methacrylamide hydrochloride (NAPMA), 3-(glycidyloxypropyl)trimetoxysilane (GOPTS), glutaraldehyde (GA), acetone, 1-ethyl-3-(3-dimethylaminopropyl)-carbodiimide hydrochloride (EDC), *N*-hydroxysuccinimide (NHS), GlcNAc, ConA, 3-aminopropyltriethyloxysilane (APTES), bis(triethoxysilyl)ethane (BTSE), *N*-ethyldiisopropylamine, sodium cyanoborohydride were purchased from Sigma-Aldrich, UK. 2-Aminoethyl-alfa-mannopyranoside was obtain from Broadpharm, USA and 2,2,2-trifluoroethyl acrylate from Thermo Fisher, USA. Phosphate buffered saline (PBS) pH 7.4 was prepared, as specified, from PBS buffer tablets (Sigma-Aldrich, UK) and consisted of phosphate buffer (0.01 M), potassium chloride (0.0027 M), and sodium chloride (0.137 M). Glass beads (Spheriglass 2429 CP00, 53–106 μm diameter) were purchased from Blagden Chemicals, UK. In all experiments double-distilled ultrapure (DI) water was used. All chemicals and solvents were of analytical or HPLC grade and used without further purification.

### Preparation of man-modified glass beads (solid-phase)

Glass beads were first activated by boiling in a 5 M NaOH for 30 min, and then washed with deionized water (five times). Beads are then incubated in 50% v/v solution of sulfuric acid for one hour, washed with deionized water (twice), and incubated in 10 mM PBS buffer for 5 min. The beads are washed with deionized water (until a pH of 7.5 was measured on the wash solution) then rinsed with acetone (twice), dried at 150 °C for 30 min and subsequently incubated overnight in a mixture of 4% v/v 3-aminopropyltriethyloxysilane (Sigma Aldrich) and 0.2% 1,2-bis(triethoxysilyl)ethane in dry toluene (0.4 mL solution per g glass beads). Afterwards, beads are washed with ten volumes of distilled water and dried in the over at 150 °C for 90 min.

Then 100 g of glass beads were placed in a solution of 7% v/v glutaric dialdehyde in 10 mM PBS for two hours, washed with deionized water (eight times). Subsequently, glass beads were incubated in 0.5 mg mL^−1^ 2-aminoethyl-alfa-mannopyranoside in 10 mM PBS for two hours. For quenching of the reaction ethanolamine hydrochloride (0.1 mg mL^−1^) was added to the beads, together with sodium cyanoborohydride (1 mg mL^−1^ in 10 mM PBS) for 30 min. Eventually, glass beads were washed with deionized water (fourteen times) and dried under vacuum.

### Preparation of GlcNAc-modified glass beads (solid-phase)

The same steps as in the preparation of Man-modified glass beads were followed however, after activation, the glass beads were incubated in a mixture of 2% v/v of 3-glycidyloxypropyl trimethoxysilane and 200 μL of 1,2-bis(triethoxysilyl)ethane in anhydrous toluene, containing also *N*-ethyldiisopropylamine 2 mg mL^−1^, at 70 °C for 24 h. Afterwards, the glass beads were rinsed with toluene twice, then with ethanol (three times), and finally acetone (five times) and dried under vacuum.

These epoxy-functionalised beads were then incubated in a solution of GlcNAc at 10 mg mL^−1^ in potassium chloride/sodium hydroxide buffer pH = 12.5 for three hours. Finally, the glass beads were rinsed with deionized water (six times), then twice with deionized water at 50 °C, twice with ethanol, and finally dried under vacuum.

### Synthesis of fluorescent nanoMIPs

For the synthesis of Man and GlcNAc nanoMIPs, the following monomers were dissolved in acetonitrile (12 g): PETMP (0.18 g), MAA (2.88 g), EGDMA (3.24 g), TRIM (3.24 g), 2,2,2-trifluoroethylacrylate (0.5 g), NAPMA (6.5 mg), *N*-fluoresceinyl acrylate (15 mg) and 0.75 g of INIFERTER. NAPMA was previously dissolved in water (1 mL) and then added to the polymeric mixture. The solution containing the monomers was degassed by purging with 20 min with N_2_. Then, 30 g of template-derivatised glass beads were added to the solution. The polymerisation was accomplished under UV radiation (1 min and 45 s). Unreacted monomers were removed by washing ten times with ACN (RT). High affinity nanoMIPs were collected by washing five times with 20 mL hot ACN (60 °C). To remove unreacted monomers, nanoMIPs were placed in a spectra/Por dialysis membrane (MWCO 10 kDa) and dialysed against deionised water for 5 days.

### Size and concentration analysis of nanoMIPs

Particle size was measured with a Zetasizer Nano (Nano-S) particle-size analyser from Malvern Instruments Ltd (UK). An aliquot of the dispersion of nanoMIPs in distilled water was sonicated for 15 min, vortexed for 30 min and then analysed by DLS at 25 °C in a 3 mL disposable polystyrene cuvette. Attenuator position, measurement duration and number of runs were automatically chosen by the instrument. The values are reported as an average of 6 measurements. The nanoMIP concentration was calculated from the absorbance intensity (at *λ*-198 nm), based on a previously obtained calibration curve.

### SPR analysis

Experiments were performed on SIA Au SPR gold chips (GE Healthcare) modified with mercaptoundecanoic acid. To achieve this, bare gold chips were first cleaned by hydrogen plasma at 50 W during five min on an Emitech K1050X Plasma Cleaner (Emitech) and then placed in ethanol containing 2.2 mg mL^−1^ mercaptoundecanoic acid overnight. After surface modification, chips were rinsed with ethanol and dried under a stream of N_2_, assembled on the holder following the manufacturer's instructions and docked onto the SPR instrument (Biacore 3000, GE Healthcare). An immobilisable version of the target (2-aminoethyl-alfa-mannopyranoside) was immobilised *via* EDC/NHS chemistry. In particular, chips were activated by injection of 100 μL EDC 0.2 M and NHS 0.5 M in water at 5 μL min^−1^ prior to template immobilisation, followed by injection of ethanolamine (0.1 M) to block any residual NHS esters. GlcNAc was immobilised on a cysteamine-modified chip (bearing primary amine groups) *via N*,*N*′-disuccinimidyl carbonate chemistry. Three control channels were used to account for non-specific binding: one was left unmodified, another was passivated with ethanolamine, whilst the last one was modified with aspartic acid. Once a stable baseline was obtained, Man nanoMIPs were injected at concentrations ranging from 20 nM down to 0.08 nM. The analysis was performed in PBS at pH 7.4 at 30 μL min^−1^. Kinetic analysis of the sensorgrams was performed with the BiaEvaluation software v4.1 assuming a 1 : 1 Langmuir binding model with baseline drifting.

### Flow cytometry

Bacteria (OD 0.3–2.4 × 10^8^ CFU mL^−1^) were washed with sterile 10 mM PBS buffer (pH 7) three times to remove residual monosaccharides which are contained in medium. Washed bacteria were incubated in 2% v/v paraformaldehyde in PBS for 30 min, then washed with PBS three times. Bacteria were then incubated in 10 mM ethanolamine for 20 min, washed with 10 mM PBS three times and finally incubated with nanoMIPs for two hours. Binding was detected using a BD Accuri C6 Plus (BD Biosciences) flow cytometer to measure nanoMIPs-associated fluorescence. Before measuring, samples were diluted ten times with PBS.

### Confocal microscopy

Slides were first modified with amino groups according to the following protocol: microscopy slides were first activated by boiling in a 5 M NaOH for 30 min. The slides were then washed with deionized water (five times), incubated in 50% v/v solution of sulfuric acid for one hour, and washed with deionized water (twice), until a pH of 7.5 was measured on the wash solution. The slides were then dried under N_2_ and subsequently incubated overnight in a mixture of 2% v/v 3-aminopropyltriethyloxysilane (Sigma Aldrich) and 0.4% of 1,2-bis(triethoxysilyl)ethane in dry toluene solution. Bacteria were then first fixed by 2% v/v paraformaldehyde and then incubated with nanoMIPs in PBS for two hours. After incubation, EDC (10 mg) and NHS (15 mg) were added and immediately applied on modified microscopic slides and left incubated for 20 min. Afterwards, slides were washed three times with PBS, 10 mM ethanolamine was added and left another 20 min. Slides were then washed again, and DAPI (1 μg mL^−1^) in PBS was added for 15 min. Subsequently, slides were washed with PBS and fixed with cover slides. Images were taken using an Olympus FV1000 confocal laser scanning microscope with a 60× oil objective using a 488 nm laser and HSD detector.

### ConA labelling

ConA (0.8 mg mL^−1^) was incubated in 10 mM PBS (pH 7.4) with 60 μL of NHS-Alexa Fluor 647 (0.5 mg mL^−1^) for 60 min. The mixture was then washed four times in a 10 kDa Amicon centrifugal unit with 10 mM PBS (pH 7) to remove unreacted dye.

### Preparation of bacteria

Two different bacteria including Gram positive *S. pneumoniae* and Gram-negative *E. coli* were used for testing of nanoMIPs selectivity. *S. pneumoniae* strains (D39, Serotype 2 strain, Laboratory collection) were grown in brain heart infusion (BHI) broth, or on blood agar plates supplemented with 5% (v/v) defibrinated horse blood at 37 °C. *E. coli* strain (One Shot TOP10 Chemically Competent *E. coli* – Thermo Fisher) was grown in Luria broth (LB) or on Luria broth agar at 37 °C and 150 rpm. The concentrations of bacterial solutions were determined by optical density at 600 nm and were adjusted to a concentration ∼1 × 10^8^ CFU mL^−1^.

## Conclusion

A novel approach based on detection of pathogenic surface saccharides by nanoMIPs was investigated. Fluorescent nanoMIPs were prepared by means of a solid-phase imprinting approach, in which two different monosaccharides (Man and GlcNAc) were used as templates. The produced nanoMIPs showed a homogeneous size distribution between 60 and 110 nm, and a nanomolar dissociation constant as measured by SPR. Thanks to their fluorescent feature endowed by the fluorescent monomer used in the polymerisation process, the nanoMIPs were successfully used for pathogen visualization, both by confocal microscopy and flow cytometry. We have demonstrated for the first time that nanoMIPs produced against bacterial surface saccharides can be used as detection or imaging tools in bacteria, both against Gram-positive (*S. pneumoniae*) and Gram-negative (*E. coli*) pathogens.

Collectively, these results demonstrate that the synthesized nanoMIPs are able to specifically recognise bacterial surface monosaccharides. These nanoMIPs can potentially serve as synthetic lectins in diagnostics. Specific recognition of rare bacterial sugars (*e.g.*, the 4,6-dideoxy sugar anthrose which is distinctive of *Bacillus anthracis*^29^) can serve to typify strains or can be used for the study of bacterial glycome. This proof-of-concept work can open up many other avenues of research, such as mapping of pathogenic surfaces, pathogenic detection and quantification, as well as investigation of pathogen and host–cell interactions, and the formation of bacterial microcolonies and biofilms. Furthermore, the developed nanoMIPs can also potentially be used to bind and block pathogenic sugars in order to influence pathogen vitality.

## Author contributions

JB: methodology, investigation, writing original. FC: conceptualisation, methodology, writing/editing, FG: methodology, writing/editing, HY: investigation, MV: supervision, writing/editing, SP: supervision, funding acquisition.

## Conflicts of interest

There are no conflicts to declare.

## Supplementary Material

NA-005-D2NA00913G-s001
