# Supplementary material for: Molecularly imprinted nanoparticles for pathogen visualisation
Source: Nanoscale Adv. 2023 Mar 17;5(9):2602–9. doi: 10.1039/d2na00913g (PMC10153071; doi:10.1039/d2na00913g)
Supplement: NA-005-D2NA00913G-s001 [file NA-005-D2NA00913G-s001.pdf]

Electronic Supplementary Material (ESI) for Nanoscale Advances.
This journal is © The Royal Society of Chemistry 2023

MOLECULARLY IMPRINTED NANOPARTICLES FOR BACTERIAL VISUALIZATION

Jaroslava Bezdekova[1], Francesco Canfarotta*[2], Fabiana Grillo*[3], Hasan Yesilkaya[3], Marketa Vaculovicova[1], Sergey Piletsky[3]

[1] Mendel University in Brno, Zemedelska 1, Brno 613 00, Czech Republic

[2] MIP Discovery, The Exchange, Colworth Science Park, Sharnbrook, MK44 1LQ, UK

[3] University of Leicester, University Rd, Leicester LE1 7RH, UK

*Corresponding author

Supplement information

Tables

Table S1 Overview of publications on bacterial detection/targeting (information are obtained from www.webofknowledge.com - Key words: imprint* polymer*, bacteria) – imprinting of whole bacteria

Whole bacteria imprinting

**Bacteria**	**Year**	**Method**	**Detection**	**Ref.**
Staphylococcus aureus	2020	Bulk polymerization	Fluorescence microscopy	[1]
Escherichia coli	2020	Microcontact imprinting	SPR	[2]
Escherichia coli	2020	Bulk polymerization	Fluorescence spectrometry	[3]
Acinetobacter baumannii	2020	Electropolymerization	Impedance spectroscopy	[4]
Vibrio parahaemolyticus	2020	Microcontact imprinting	PCR/gel electrophoresis	[5]
Escherichia coli, Escherichia blattae	2020	Microcontact imprinting	Pyroelectric detection	[6]
Escherichia coli	2019	Soft-lithography	Thermal resistance	[7]
Escherichia coli	2019	Microcontact imprinting	Time-dependent temperature measurement	[8]
Listeria Monocytogenes	2019	Pickering emulsion polymerization	Fluorescence microscopy	[9]
Enterococcus faecalis	2019	Emulsion polymerization	SPR	[10]
Escherichia coli	2019	Sol-gel imprinting	Impedimetric	[11]
Escherichia coli	2019	Electropolymerization	Impedimetric	[12]
Escherichia coli	2018	Microcontact imprinting	Thermal	[13]
Escherichia coli	2018	Nanoimprint lithography	Fluorescent microscopy	[14]
Escherichia coli, Listeria monocytogenes	2018	Sol–gel imprinting	Fluorescence spectrometry	[15]

Escherichia coli	2017	Electropolymerization	QCM	[16]
Escherichia coli	2017	Soft-lithography	Thermal	[17]
Staphylococcus epidermidis	2017	Electropolymerization	Impedimetric	[18]
Escherichia coli	2017	Soft-lithography	Heat-transfer method	[19]
Escherichia coli, Shewanella oneidensis, Staphylococcus aureus, Enterococcus faecium	2017	Atomic transfer radical polymerization	Single-cell force spectroscopy	[20]
Escherichia coli	2017	Bulk polymerization	Cyclic voltammetry	[21]
Escherichia coli	2017	Microcontact imprinting	Cyclic voltammetry	[22]
Escherichia coli	2017	Nanoimprint lithography	QCM	[23]
Escherichia coli, Staphylococcus aureus	2016	Microcontact imprinting	Heat-transfer method	[24]
Escherichia coli	2016	Sedimentation/stamp imprinting	QCM	[25]
Bacillus Cereus	2016	Sedimentation/stamp imprinting	QCM	[26]
Rhodobacter sphaeroides	2015	Microcontact imprinting	Raman spectrometry	[27]
Escherichia coli	2015	Microcontact imprinting	SPR/QCM	[28]
Escherichia coli, Staphylococcus aureus	2014	Imprinted shells	Fluorescence microscopy	[29]
Methylomicrobium album, Methylosinus trichosporium	2014	Stamp imprinting	Fluorescence microscopy	[30]
Escherichia coli, Micrococcus luteus	2014	Pickering emulsion polymerization	Fluorescence microscopy	[31]
Escherichia coli, Pseudomona aeruginosa, Bacillus subtilis, Staphylococcus aureus	2014	Electropolymerization	Dielectrophoresis	[32]
Mycobacterium tuberculosis	2013	Sedimentation imprinting	Fluorescence microscopy	[33]
Bacillus subtilis	2013	Electropolymerization	Impedimetric	[34]
Escherichia coli, Klebsiella pneumoniae, Staphylococcus aureus, Staphylococcus epidermidis	2012	Soft lithography	Confocal microscopy	[35]
Synechococcus elongatus	2012	Soft lithography	Inverted microscopy	[36]

Table S2 Overview of publications on bacterial detection/targeting (information are obtained from www.webofknowledge.com - Key words: imprint* polymer*, bacteria) – imprinting of proteins or epitopes

Epitope/ Protein imprinting

Bacteria	Target	Year	Method	Detection	Ref.
Mycobacterium leprae	Peptide	2019	Electropolymerization	QCM	[37]
Neisseria Meningitidis	Peptide	2018	Bulk polymerization	QCM	[38]
Staphylococcus aureus	Surface protein	2016	Electropolymerization	Cyclic voltammetry	[39]
Pseudomonas aeruginosa	Lipopoly-saccharide	2016	Precipitate polymerization	Fluorescence polarization	[40]
Staphylococcus aureus	Surface protein	2009	Bulk polymerization	UV spectrophotometry	[41]

Table S3 Summary of publications focused on the imprinted polymers of saccharides

Saccharide	Cell	Year	Ref.
Sialic acid	DU145 cell, PC3 cell, Jurkat cell	2015	[42]
Glucuronic acid	Human keratinocytes	2015	[43]
Sialic acid, Fucose, Mannose	HepG-2 cell, L-02 cell, MCF-7 cell, MCF-10A cell	2016	[44]
Sialic acid	DU 145 cell, HeLa cell	2017	[45]
Hyaluronan, Sialic acid	HaCaT cell	2017	[46]
Hyaluronan	HaCaT cell	2017	[47]
Hyaluronic acid	HaCaT cell	2018	[48]
Hyaluronic acid	HaCaT cell	2019	[46]
Sialic acid, Fucose, Mannose	HepG-2 cell, L-02 cell, MCF-10A cell, A-431 cell, HaCat cell, HK-2 cell, HeLa cell	2017	[49]

Results

Dynamic light scattering (DLS) analysis was performed on nanoMIPs after 2 min sonication and 30 min vortexing. The data show a single peak, indicating an average hydrodynamic diameter of 55.56 ± 6.1 nm for Man nanoMIPs (a) and 111 ± 7.5 for GlcNAc nanoMIPs (b).

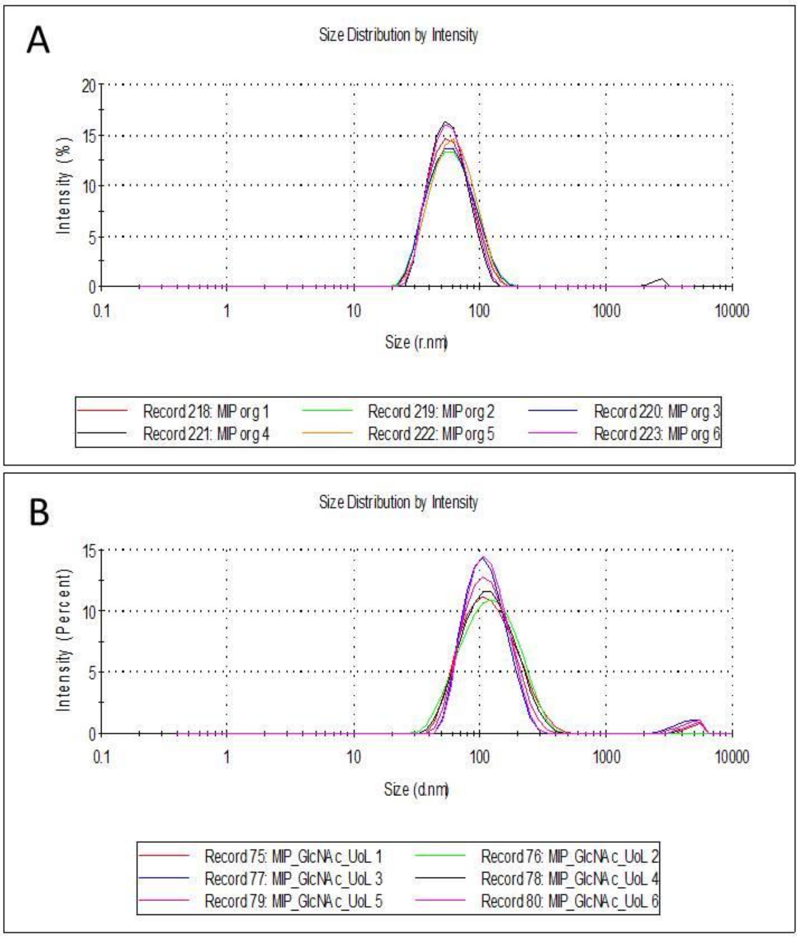

Figure S1 DLS analysis of Man-nanoMIPs (a), GlcNAc-nanoMIPs (b).

Flow cytometry analysis

Different concentrations of GlcNAc-nanoMIPs (0.00015 - 0.40000 mg/mL) and Man-nanoMIPs (0.0125 - 0.40000 mg/mL) were incubated respectively *E.Coli* (Figure S2) and *D39_S. Pneumoniae* (Figure S3). In the graphs reported below, the shift in the fluorescent signal indicates binding between fluorescent nanoMIPs and respectively *E.Coli* or *D39_S.Pneumoniae*. Both bacteria were run without nanoMIPs and the autofluorescence subtracted. NanoMIPs imprinted for a small non-related compound were incubated with both bacteria and used as controls.

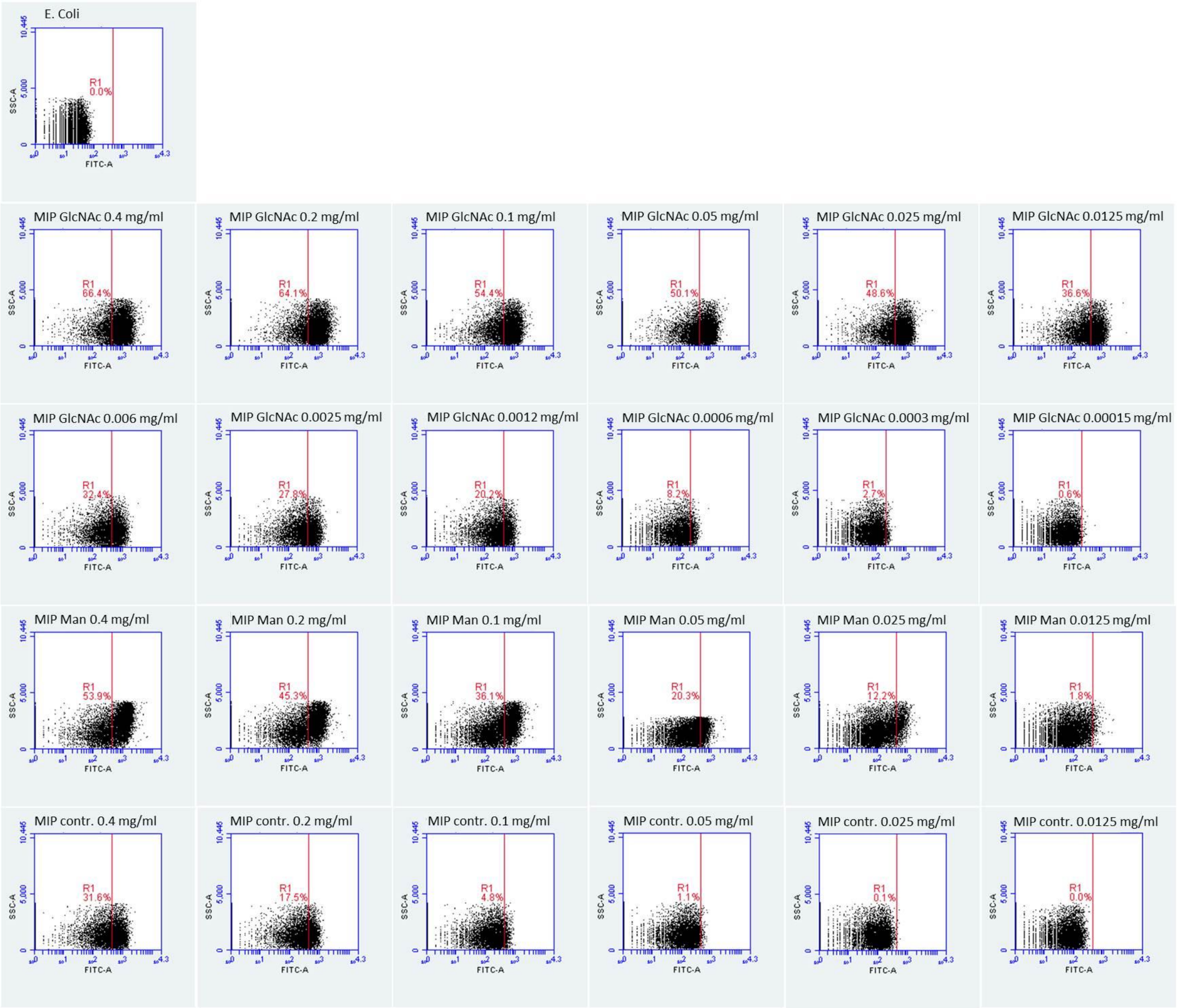

Figure S2 Flow cytometry analysis of control *E. Coli (no nanoMIPs)*, GlcNAc nanoMIPs (0.00015 – 0.40000 mg/mL) binding *E. Coli;* Man NanoMIPs (0.00125 – 0.4000 mg/mL) binding *E.Coli* and control nanoMIPs binding *E.Coli*

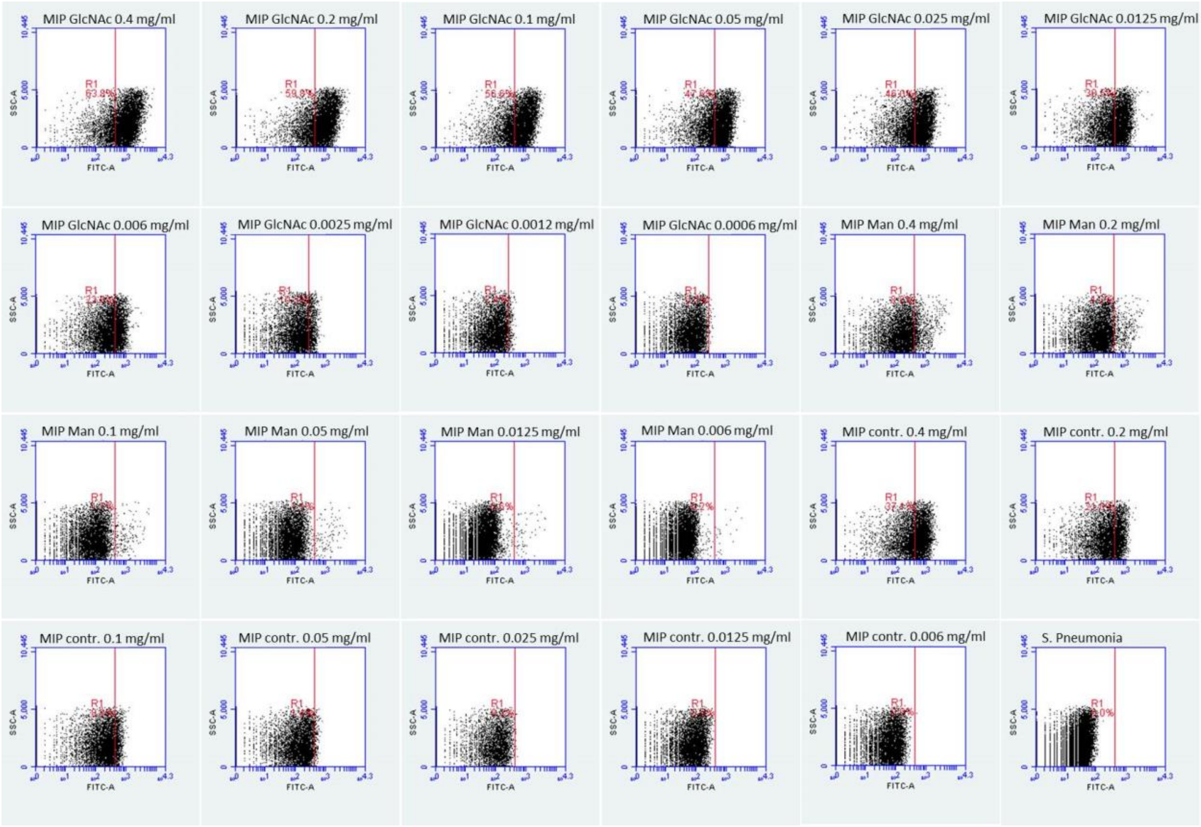

Figure S3 Flow cytometry analysis of GlcNAc nanoMIPs (0.0006 – 0.4 mg/mL) binding *S. Pneumoniae;* Man NanoMIPs (0.006 – 0.4 mg/mL) binding *S. Pneumoniae*; control nanoMIPs binding *S. Pneumoniae; Control S. Pneumoniae (no nanoMIPs)*

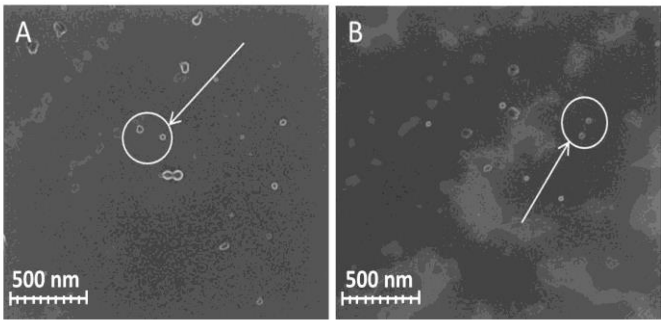

Figure S4 (A) Image of Man-nanoMIPs by SEM; (B) image of GlcNAc-nanoMIPs by SEM.

Comparison of binding

ConA was labelled with a fluorescent dye (Alexa 647) and binding between bacteria and ConA was assessed by using flow cytometry. From measured data (Fig S.4) it was observed that the amount of bound ConA was higher in the case of *E. Coli*. These data confirm that *E. Coli* bears higher amount of mannose molecule on their surface compared with *S. Pneumoniae*.

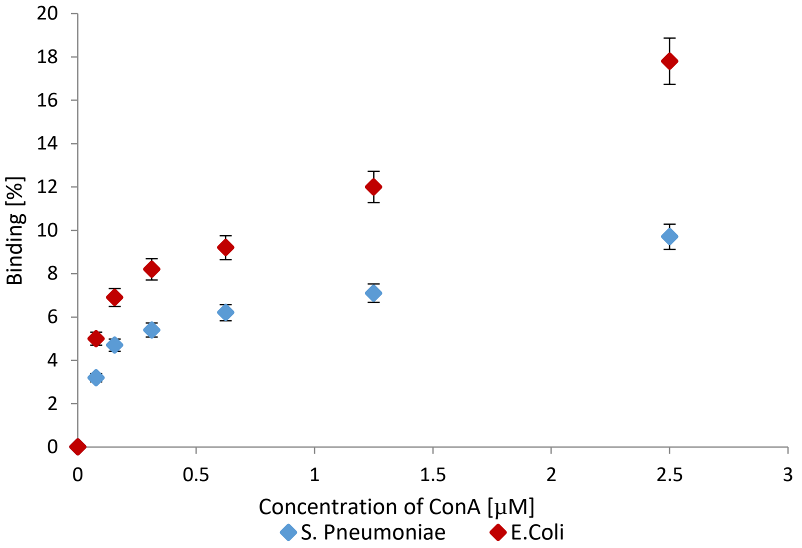

Figure S5 Binding of concanavalin A to gram-negative (red) and positive (blue) bacteria by flow cytometry.

1. Bezdekova, J., et al., *Magnetic molecularly imprinted polymers used for selective isolation and detection of Staphylococcus aureus.* Food Chemistry, 2020: p. 126673.
2. Özgür, E., et al., *Surface plasmon resonance based biomimetic sensor for urinary tract infections.* Talanta, 2020. **212**: p. 120778.
3. Mankar, J.S., et al., *Molecularly imprinted microparticles (microMIPs) embedded with reduced graphene oxide for capture and destruction of E. coli in drinking water.* Materials Science and Engineering: C, 2020: p. 110672.
4. Roushani, M., M. Sarabaegi, and A. Rostamzad, *Novel electrochemical sensor based on polydopamine molecularly imprinted polymer for sensitive and selective detection of Acinetobacter baumannii.* JOURNAL OF THE IRANIAN CHEMICAL SOCIETY, 2020.
5. Fu, K., et al., *Rapid and selective recognition of Vibrio parahaemolyticus assisted by perfluorinated alkoxysilane modified molecularly imprinted polymer film.* RSC Advances, 2020. **10**(24): p. 14305-14312.
6. Givanoudi, S., et al., *An imaging study and spectroscopic curing analysis on polymers for synthetic whole-cell receptors for bacterial detection.* Japanese Journal of Applied Physics, 2020. **59**(SD): p. SD0802.
7. Cornelis, P., et al., *Sensitive and specific detection of E. coli using biomimetic receptors in combination with a modified heat-transfer method.* Biosensors and Bioelectronics, 2019. **136**: p. 97-105.
8. Heidt, B., et al., *Biomimetic Bacterial Identification Platform Based on Thermal Transport Analysis Through Surface Imprinted Polymers: From Proof of Principle to Proof of Application.* physica status solidi (a), 2019. **216**(12): p. 1800688.
9. Zhao, X., et al., *Preparation of fluorescent molecularly imprinted polymers via pickering emulsion interfaces and the application for visual sensing analysis of Listeria Monocytogenes.* Polymers, 2019. **11**(6): p. 984.
10. Erdem, Ö., et al., *Molecularly imprinted nanoparticles based plasmonic sensors for real-time Enterococcus faecalis detection.* Biosensors and Bioelectronics, 2019. **126**: p. 608-614.
11. Jafari, H., et al., *Entrapment of uropathogenic E. coli cells into ultra-thin sol-gel matrices on gold thin films: A low cost alternative for impedimetric bacteria sensing.* Biosensors and Bioelectronics, 2019. **124**: p. 161-166.
12. Wu, J., et al., *Facile preparation of a bacteria imprinted artificial receptor for highly selective bacterial recognition and label-free impedimetric detection.* Analytical Chemistry, 2018. **91**(1): p. 1027-1033.
13. van Grinsven, B., et al., *SIP ‐Based Thermal Detection Platform for the Direct Detection of Bacteria Obtained from a Contaminated Surface.* physica status solidi (a), 2018. **215**(15): p. 1700777.
14. Dulay, M.T., et al., *Pathogen-imprinted organosiloxane polymers as selective biosensors for the detection of targeted E. coli.* C—Journal of Carbon Research, 2018. **4**(2): p. 29.
15. Cai, W., et al., *Bacteria assisted protein imprinting in sol–gel derived films.* Analyst, 2018. **143**(2): p. 555-563.
16. Tokonami, S., et al., *Mechanism in external field-mediated trapping of bacteria sensitive to nanoscale surface chemical structure.* Scientific reports, 2017. **7**(1): p. 1-12.
17. Stilman, W., et al., *Optimization and characterization of a flow cell for heat ‐transfer ‐based biosensing.* physica status solidi (a), 2017. **214**(9): p. 1600758.

18. Golabi, M., et al., *Electrochemical bacterial detection using poly (3-aminophenylboronic acid)-based imprinted polymer.* Biosensors and Bioelectronics, 2017. **93**: p. 87-93.
19. Steen Redeker, E., et al., *Biomimetic bacterial identification platform based on thermal wave transport analysis (TWTA) through surface-imprinted polymers.* ACS infectious diseases, 2017. **3**(5): p. 388-397.
20. Bao, H., et al., *Bacteria-templated fabrication of a charge heterogeneous polymeric interface for highly specific bacterial recognition.* Chemical Communications, 2017. **53**(15): p. 2319-2322.
21. Chen, S., et al., *Electrochemiluminescence detection of Escherichia coli O157: H7 based on a novel polydopamine surface imprinted polymer biosensor.* Acs Applied Materials & Interfaces, 2017. **9**(6): p. 5430-5436.
22. Idil, N., et al., *Whole cell based microcontact imprinted capacitive biosensor for the detection of Escherichia coli.* Biosensors and Bioelectronics, 2017. **87**: p. 807-815.
23. Poller, A.-M., et al., *Surface imprints: Advantageous application of ready2use materials for bacterial quartz-crystal microbalance sensors.* Acs Applied Materials & Interfaces, 2017. **9**(1): p. 1129-1135.
24. van Grinsven, B., et al., *Label-free detection of Escherichia coli based on thermal transport through surface imprinted polymers.* ACS sensors, 2016. **1**(9): p. 1140-1147.
25. Schnettelker, A. and P. Lieberzeit, *A self-organisation synthesis approach for bacteria molecularly imprinted polymers.* Procedia Engineering, 2016. **168**: p. 557-560.
26. Spieker, E. and P.A. Lieberzeit, *Molecular imprinting studies for developing qcm-sensors for bacillus cereus.* Procedia Engineering, 2016. **168**: p. 561-564.
27. Lee, M.-H., et al., *Recognition of Rhodobacter sphaeroides by microcontact-imprinted poly (ethylene-co-vinyl alcohol).* Colloids and Surfaces B: Biointerfaces, 2015. **135**: p. 394-399.
28. Yilmaz, E., et al., *Whole cell imprinting based Escherichia coli sensors: A study for SPR and QCM.* Sensors and Actuators B: Chemical, 2015. **209**: p. 714-721.
29. Zhang, Z., et al., *Cell‐Imprinted Antimicrobial Bionanomaterials with Tolerable Toxic Side Effects.* Small, 2015. **11**(11): p. 1258-1264.
30. Hu, Y., et al., *Isolation of viable type I and II methanotrophs using cell-imprinted polyurethane thin films.* Acs Applied Materials & Interfaces, 2014. **6**(22): p. 20550-20556.
31. Shen, X., et al., *Bacterial imprinting at Pickering emulsion interfaces.* Angewandte Chemie International Edition, 2014. **53**(40): p. 10687-10690.
32. Tokonami, S., et al., *Recognition of gram-negative and gram-positive bacteria with a functionalized conducting polymer film.* Research on Chemical Intermediates, 2014. **40**(6): p. 2327-2335.
33. Ren, K., N. Banaei, and R.N. Zare, *Sorting inactivated cells using cell-imprinted polymer thin films.* Acs Nano, 2013. **7**(7): p. 6031-6036.
34. Qi, P., Y. Wan, and D. Zhang, *Impedimetric biosensor based on cell-mediated bioimprinted films for bacterial detection.* Biosensors and Bioelectronics, 2013. **39**(1): p. 282-288.
35. Ren, K. and R.N. Zare, *Chemical recognition in cell-imprinted polymers.* Acs Nano, 2012. **6**(5): p. 4314-4318.
36. Schirhagl, R., et al., *Separation of bacteria with imprinted polymeric films.* Analyst, 2012. **137**(6): p. 1495-1499.
37. Kushwaha, A., et al., *Epitope imprinting of Mycobacterium leprae bacteria via molecularly imprinted nanoparticles using multiple monomers approach.* Biosensors and Bioelectronics, 2019. **145**: p. 111698.
38. Gupta, N., et al., *Epitope imprinting of iron binding protein of Neisseria meningitidis bacteria through multiple monomers imprinting approach.* Journal of Molecular Recognition, 2018. **31**(7): p. e2709.
39. Khan, M.A.R., et al., *Plastic antibody for the electrochemical detection of bacterial surface proteins.* Sensors and Actuators B: Chemical, 2016. **233**: p. 697-704.
40. Long, Y., et al., *Novel polymeric nanoparticles targeting the lipopolysaccharides of Pseudomonas aeruginosa.* International journal of pharmaceutics, 2016. **502**(1-2): p. 232-241.
41. Pan, J., et al., *Recognition property and preparation of Staphylococcus aureus protein A-imprinted polyacrylamide polymers by inverse-phase suspension and bulk polymerization.* Polymer, 2009. **50**(11): p. 2365-2372.
42. Shinde, S., et al., *Sialic acid-imprinted fluorescent core–shell particles for selective labeling of cell surface glycans.* Journal of the American Chemical Society, 2015. **137**(43): p. 13908-13912.
43. Kunath, S., et al., *Cell and tissue imaging with molecularly imprinted polymers as plastic antibody mimics.* Advanced healthcare materials, 2015. **4**(9): p. 1322-1326.
44. Wang, S., et al., *Targeting and imaging of cancer cells via monosaccharide-imprinted fluorescent nanoparticles.* Scientific reports, 2016. **6**: p. 22757.
45. Liu, R., et al., *Preparation of sialic acid-imprinted fluorescent conjugated nanoparticles and their application for targeted cancer cell imaging.* Acs Applied Materials & Interfaces, 2017. **9**(3): p. 3006-3015.
46. Demir, B., et al., *Tracking hyaluronan: molecularly imprinted polymer coated carbon dots for cancer cell targeting and imaging.* Acs Applied Materials & Interfaces, 2018. **10**(4): p. 3305-3313.
47. Panagiotopoulou, M., et al., *Fluorescent molecularly imprinted polymers as plastic antibodies for selective labeling and imaging of hyaluronan and sialic acid on fixed and living cells.* Biosensors and Bioelectronics, 2017. **88**: p. 85-93.
48. Poma, A., et al., *Solid‐phase synthesis of molecularly imprinted polymer nanoparticles with a reusable template–"plastic antibodies"*. Advanced functional materials, 2013. **23**(22): p. 2821-2827.
49. Wang, S., et al., *Pattern recognition of cells via multiplexed imaging with monosaccharide-imprinted quantum dots.* Analytical Chemistry, 2017. **89**(10): p. 5646-5652.
